# Quantification of Ebola virus replication kinetics in vitro

**DOI:** 10.1371/journal.pcbi.1008375

**Published:** 2020-11-02

**Authors:** Laura E. Liao, Jonathan Carruthers, Sophie J. Smither, CL4 Virology Team, Simon A. Weller, Diane Williamson, Thomas R. Laws, Isabel García-Dorival, Julian Hiscox, Benjamin P. Holder, Catherine A. A. Beauchemin, Alan S. Perelson, Martín López-García, Grant Lythe, John N. Barr, Carmen Molina-París

**Affiliations:** 1 Theoretical Biology and Biophysics, Los Alamos National Laboratory, Los Alamos, NM, USA 87545; 2 Department of Applied Mathematics, School of Mathematics, University of Leeds, Leeds LS2 9JT, UK; 3 Defence Science and Technology Laboratory, Salisbury SP4 0JQ, UK; 4 Institute of Infection and Global Health, University of Liverpool, Liverpool, L69 7BE, UK; 5 Department of Physics, Grand Valley State University, Allendale, MI, USA 49401; 6 Department of Physics, Ryerson University, Toronto, ON, Canada M5B 2K3; 7 Interdisciplinary Theoretical and Mathematical Sciences (iTHEMS) Research Program at RIKEN, Wako, Saitama, Japan, 351-0198; 8 School of Molecular and Cellular Biology, University of Leeds, Leeds LS2 9JT, UK; The University of Melbourne Melbourne School of Psychological Sciences, AUSTRALIA

## Abstract

Mathematical modelling has successfully been used to provide quantitative descriptions of many viral infections, but for the Ebola virus, which requires biosafety level 4 facilities for experimentation, modelling can play a crucial role. Ebola virus modelling efforts have primarily focused on *in vivo* virus kinetics, e.g., in animal models, to aid the development of antivirals and vaccines. But, thus far, these studies have not yielded a detailed specification of the infection cycle, which could provide a foundational description of the virus kinetics and thus a deeper understanding of their clinical manifestation. Here, we obtain a diverse experimental data set of the Ebola virus infection *in vitro*, and then make use of Bayesian inference methods to fully identify parameters in a mathematical model of the infection. Our results provide insights into the distribution of time an infected cell spends in the eclipse phase (the period between infection and the start of virus production), as well as the rate at which infectious virions lose infectivity. We suggest how these results can be used in future models to describe co-infection with defective interfering particles, which are an emerging alternative therapeutic.

## Author summary

The two deadliest Ebola virus epidemics have both occurred in the past five years, with one of these epidemics still ongoing. Mathematical modelling has already provided insights into the spread of disease at the population level as well as the effect of antiviral therapy in Ebola virus-infected animals. However, a quantitative description of the replication cycle is still missing. Here, we report results from a set of *in vitro* experiments involving infection with the Ecran strain of Ebola virus. By parameterizing a mathematical model, we are able to determine robust estimates for the duration of the replication cycle, the infectious burst size, and the viral clearance rate.

## Introduction

The world’s second largest Ebola outbreak is currently underway in the Democratic Republic of Congo. Ebola virus (EBOV) causes severe and fatal disease with death rates of up to 90% [1]. There is an urgent need to prevent and treat EBOV infections, but no antiviral drugs or monoclonal antibodies have been approved in Africa, the EU, or the US. Recently the first EBOV vaccine has been approved by European regulators [2]. Experimental therapies [3], including antiviral drugs (remdesivir [4] and favipiravir [5, 6]) and a cocktail of monoclonal antibodies (ZMapp) [7], have been assessed in the 2013–2016 West Africa Ebola virus disease outbreak. Other promising monoclonal antibody therapies, called mAb114 and REGN-EB3, have been deployed in the current 2018–2019 Kivu Ebola virus epidemic [8]. A better understanding of the precise infection kinetics of EBOV is warranted.

Mathematical modelling of viral dynamics has provided a quantitative understanding of within-host viral infections, such as HIV [9], influenza [10], Zika [11], and more recently, EBOV. Mathematical modelling studies have analyzed the plasma viral load dynamics of EBOV-infected animals (mice [12], non-human primates [13, 14]) while under therapy with favipiravir, and have identified estimates of favipiravir efficacy and target drug concentrations. In addition, mechanistic models of innate and adaptive immune responses were used to provide an explanation of EBOV infection dynamics in non-human primates [14], and of differences between fatal and non-fatal cases of human infection [15]. Moreover, mathematical models have been used to predict the effect of treatment initiation time on indicators of disease severity [12, 15] and survival rates [14], to predict the clearance of EBOV from seminal fluid of survivors [16], and to theoretically explore treatment of EBOV-infected humans with antivirals that possess different mechanisms of action (i.e., nucleoside analog, siRNA, antibody) [15].

Alongside the progress made in understanding within-host infections, a complementary view of infection can be provided by mathematical modelling of infections at the *in vitro* level. Combined with *in vitro* time course data, mathematical models (MMs) have provided a detailed quantitative description of the viral replication cycle of influenza A virus [17, 18], SHIV [19, 20], HIV [21], and other viruses [22–26]. Such studies yield estimates of key quantities such as the basic reproductive number (defined as the number of secondary infections caused by one infected cell in a population of fully susceptible cells), half-life of infected cells, and viral burst size, which cannot be obtained directly from data [27]. In the context of *in vitro* infections, parameterized MMs have been used to predict the outcome of competition experiments between virus strains [28–30] (i.e., which strain dominates in a mixed infection), map differences in genotype to changes in phenotype [28, 30] (e.g., associate a single mutation to ten-fold faster viral production), quantify fitness differences between virus strains [31, 32] (e.g., which strain has a larger infectious burst size), quantify the contribution of different modes of transmission (cell-to-cell versus cell-free) [21], and identify the target of antiviral candidates [33] (e.g., whether a drug inhibits viral entry or viral production). One prior study [34] utilized *in vitro* infection data from the literature to estimate EBOV infection parameters, but had several parameter identifiability issues due to insufficient data.

Our goal is to obtain robust estimates of viral infection parameters that characterize the EBOV replication cycle. We follow a mathematical modelling approach that has been successfully applied in the analysis of other viral infections *in vitro* [17]. To this end, we performed a suite of *in vitro* infection assays (single-cycle, multiple-cycle, and viral infectivity decay assays) using EBOV and Vero cells, and collected detailed extracellular infectious and total virus time courses. The viral kinetic data were simulated with a multicompartment ordinary differential equation MM, and posterior distributions of the MM parameters were estimated using a Markov chain Monte Carlo (MCMC) approach. We estimate that one EBOV-infected cell spends ∼30 h in an eclipse phase before it releases infectious virions at a rate of 13/h, over its infectious lifetime of ∼83 h. The number of infectious virions produced over an infected cell’s lifetime is ∼1000, with an estimated basic reproductive number of ∼600. We also discuss challenges in collecting other types of virus dynamic data (e.g., intracellular viral RNA or cell counts).

## Results

### Ebola virus kinetics in vitro

**Fig 1:**
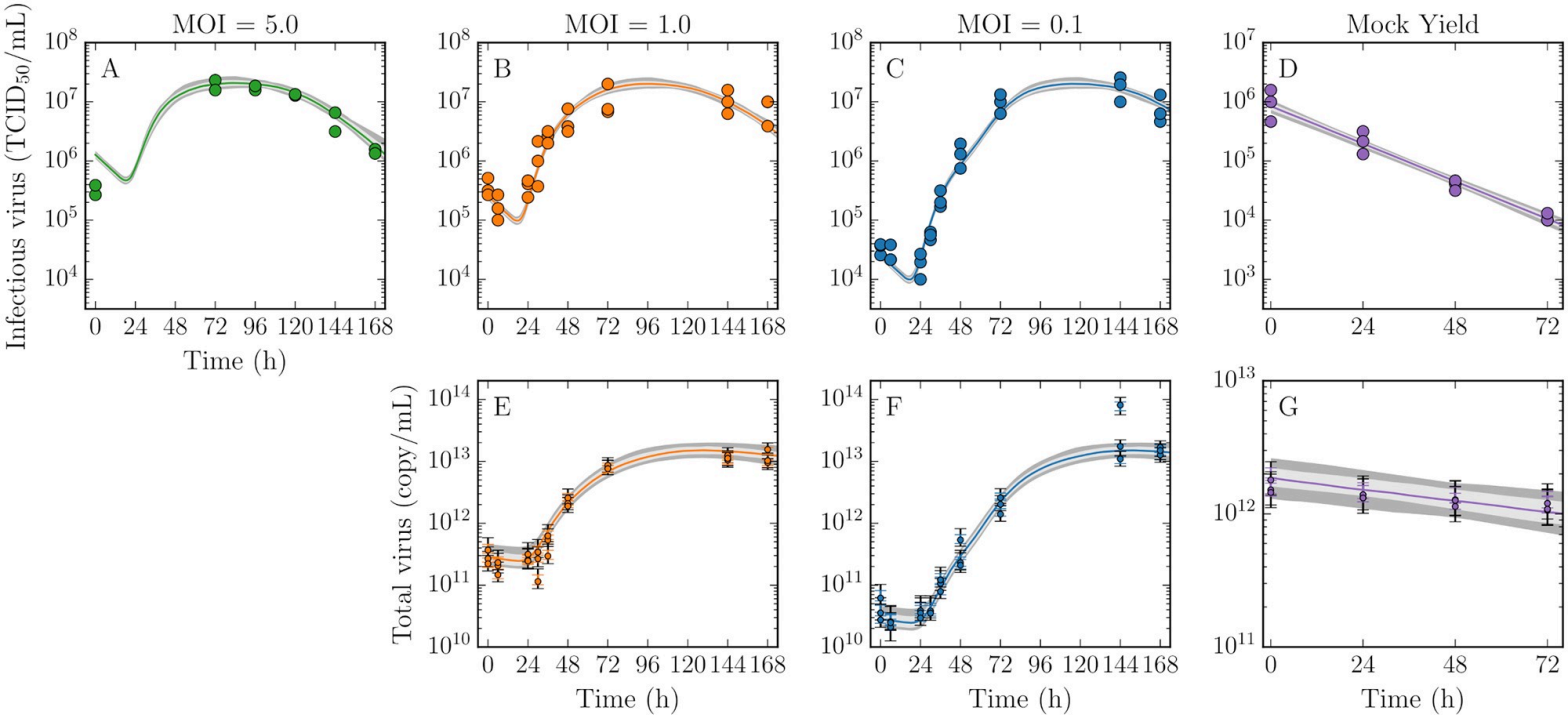
Kinetics of EBOV infection *in vitro* and mock yield assays. Vero cell monolayers were infected with EBOV at a multiplicity of infection (MOI) 5, 1, or 0.1 TCID_50_/cell, as indicated. At various times post-infection, the infectious (TCID_50_/mL; A–C) and total virus (copy/mL; E–G) in the supernatant were determined. A mock yield assay was also performed to quantify the decay of infectious (D) and total virus (G). In each assay, the experimental data (circles) were collected either in duplicate (MOI 5) or triplicate (all other assays). Note that the total virus concentration collected in the MOI 5 infection was omitted from the analysis due to inconsistencies in the peak value (S1 Appendix, Fig. A). The lines represent the pointwise median of the time courses simulated from our MM, which are bracketed by 68% (light grey) and 95% (dark grey) credible regions (CR). These data were used to extract the posterior probability likelihood distributions of the infection parameters (Fig 2). Note that parameters of the calibration curve used to convert cycle threshold values (Ct) to total virus (copy/mL) were also estimated (Fig 3). The variability introduced from this conversion is shown by two error bars on each total virus data point, indicating the 68% (same colour) and 95% (black) CR.

Vero cell monolayers were infected with EBOV at a multiplicity of infection (MOI) of 5, 1, 0.1 TCID_50_/cell. Infectious and total virus concentrations were determined from extracellular virus harvested from the supernatant of each well at various times post-infection (Fig 1A–1C, 1E and 1F). At the start of infection, the virus concentrations do not rise for some time, reflecting the time it takes for viral entry, replication and release. After 24 h, the virus concentrations grow exponentially as infected cells begin producing virus. When all cells in the well are infected, the virus concentrations peak at approximately 2 × 10^7^TCID_50_/mL and 10^13^ copy/mL and the peak is sustained for ∼72 h. Thereafter, the virus concentrations decline when virus production ceases, presumably due to the death of infected cells. Additionally, the kinetics of viral infectivity decay and virus degradation were assessed with a mock yield assay (Fig 1D and 1G). In the mock yield assay, an inoculum of virus was incubated in wells under the same conditions as the growth assays, but in the absence of cells, and sampled over time.

### Mathematical model of viral infection and parameter estimates

The *in vitro* EBOV infection kinetics were captured with a MM that has been used successfully in past works to capture influenza A virus infection kinetics *in vitro* [28, 30, 31]. The MM is given by the system of ordinary differential equations:

$$\begin{array}{cc} \frac{dT}{dt} & =-\beta TV_{\text{inf}}\\ \frac{dE_{1}}{dt} & =\beta TV_{\text{inf}}-\frac{n_{E}}{\tau_{E}}E_{1}\\ \frac{dE_{i=2,3,...,n_{E}}}{dt} & =\frac{n_{E}}{\tau_{E}}(E_{i-1}-E_{i})\\ \frac{dI_{1}}{dt} & =\frac{n_{E}}{\tau_{E}}E_{n_{E}}-\frac{n_{I}}{\tau_{I}}I_{1}\\ \frac{dI_{j=2,3,...,n_{I}}}{dt} & =\frac{n_{I}}{\tau_{I}}(I_{j-1}-I_{j})\\ \frac{dV_{\text{inf}}}{dt} & =p_{\text{inf}}\sum_{j=1}^{n_{I}}I_{j}-c_{\text{inf}}V_{\text{inf}}\\ \frac{dV_{\text{tot}}}{dt} & =p_{\text{tot}}\sum_{j=1}^{n_{I}}I_{j}-c_{\text{tot}}V_{\text{tot}} \end{array}$$ (1)

In this MM, susceptible uninfected target cells *T* can be infected by infectious virus *V*_inf_ with infection rate constant *β*, and subsequently enter the non-productive eclipse phase $E_{i=1,\ldots ,n_{E}}$, followed by a transition into the productively infectious phase $I_{j=1,\ldots ,n_{I}}$. The eclipse and infectious phases are divided into a number of compartments given by *n*_*E*_ and *n*_*I*_, respectively, such that the time spent in each phase follows an Erlang distribution with an average duration of $\tau_{E,I}\pm \frac{\tau_{E,I}}{\sqrt{n_{E,I}}}$. While cells are in the infectious phase, they produce infectious (total) virus *V*_inf_ (*V*_tot_) at a rate *p*_inf_ (*p*_tot_), which lose infectivity (viability) at rate *c*_inf_ (*c*_tot_). The MM Eq (1) captures both infectious virus, quantified by TCID_50_ measurements of supernatant samples, and total virus, quantified by quantitative, real-time, reverse transcriptase PCR (hereafter, RT-qPCR). The latter experimental quantity was obtained by converting cycle threshold (Ct) values from RT-qPCR to copy number (Fig 3) using Eq (3) (Methods).

**Fig 2:**
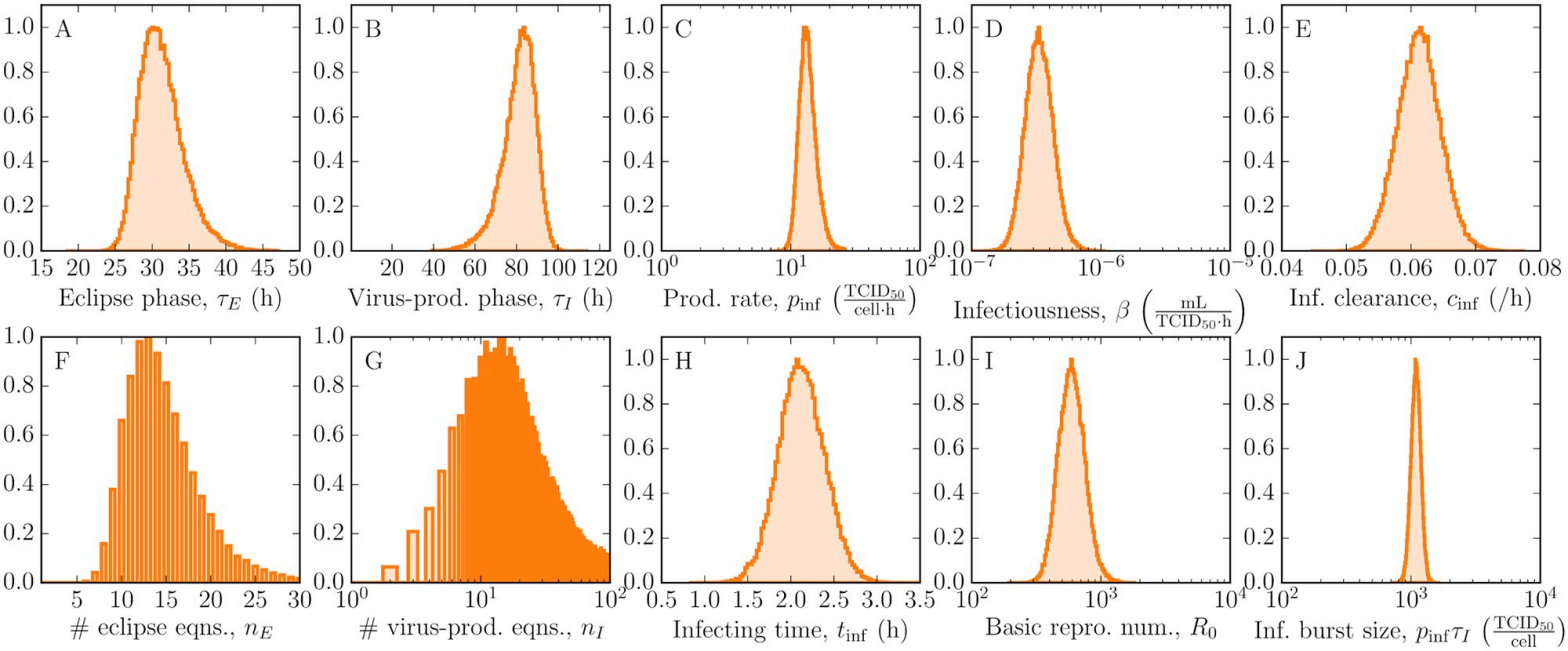
Estimated parameter distributions of EBOV infection *in vitro*. Posterior probability likelihood distributions (PostPLDs) of parameters in the MM (A–G) were estimated using MCMC and the data in Fig 1. Secondary parameters were derived from these estimates (H–J). Note that the PostPLDs corresponding to the number of eclipse and infectious phase compartments are integer-valued. The remaining PostPLDs of parameters describing the total virus and calibration curve are in S1 Appendix, Fig. B.

Predicted virus time courses from the MM are shown in Fig 1 where the solid lines represent the pointwise median and the grey bands show narrow 95% credible regions (CR), indicating that the MM reproduces the viral kinetic data well. Using a Markov chain Monte Carlo (MCMC) approach, we obtained posterior probability likelihood distributions (PostPLDs) for each of the MM parameters (Fig 2). Narrow PostPLDs were extracted with mild correlations between parameters (S1 Appendix, Fig. C), indicating good practical identification of all parameters.

### A quantitative description of the EBOV lifecycle

The MCMC analysis gives us the following quantitative description of the EBOV lifecycle within Vero cells. An EBOV-infected Vero cell spends approximately 30 h with a 95% credible region of [26 h, 37 h] in the eclipse phase before progeny EBOV successfully bud. Subsequently, infectious virus is produced at a rate of 13 [10, 20] virions per cell per hour over a duration of 83 h [64 h, 95 h] before virus production ceases due to cell death. An infectious burst size of 1096 progeny virions [1000, 1259] is released from each infected cell over its virus-producing lifetime. Once infectious virus enters the cell culture medium, they lose infectivity at a rate of 0.06 /h [0.055 /h, 0.068 /h], which is comparable to other viruses such as influenza A virus [31] or SHIV [19]. Overall, the *in vitro* spread of infection is rapid, as characterized by an infecting time of 2 h [1.6 h, 2.7 h], which is defined as the time required for a single virus-producing cell to infect one more [35]. These dynamics imply a large basic reproductive number of 589 [398, 1000], which is defined as the number of secondary infections caused by a single infected cell in a population of fully susceptible cells.

Notably, we find that the durations of both the eclipse and infectious phases follow a normal-like distribution, as given by *n*_*E*_ of 13 [8, 23] and *n*_*I*_ of 14 [3, 85]. This implies that the eclipse phase comprises a sequence of many distinct steps of short duration, without any one step lasting significantly longer than the rest. Likewise, the same interpretation applies to the infectious phase. The normal-like distribution of the eclipse phase resembles that of influenza A virus [30], but contrasts with the fat-tailed eclipse phase distribution of SHIV [20] which is likely due to a process in the phase that is longer than the rest (e.g., integration). Moreover, neither the eclipse nor infectious phase are exponentially distributed (*n* = 1) as is commonly assumed in analyses with MMs. Such an assumption has been shown to impact estimates of antiviral efficacy that are based on patterns of viral load decay under simulated therapy in HIV patients [20].

## Discussion

In this work we performed time-course Ebola virus (EBOV) infection experiments at multiple MOIs *in vitro* and applied MCMC methods to precisely parameterize a mathematical model (MM) of the infection. We extracted fundamental quantities concerning the timing and viral production of EBOV replication. This theoretical-experimental approach maximized the output of the costly and difficult experiments, which must be performed in biosafety level 4 facilities. Previous studies of the EBOV lifecycle rely on safer virus-like particles [36]. The only previously known MM of EBOV infection *in vitro* [34] is restricted in its use due to problems with parameter identifiability; specifically, the existence of strong correlations between parameters, such as the rates of virus degradation and virus production. By obtaining a more complete set of experimental observations, we have provided the first detailed quantitative characterization of EBOV infection kinetics.

Some of our estimates of timescales in the EBOV infection kinetics fill gaps in the knowledge of this virus, while others expose some tension with prior mathematical modelling work. The eclipse phase, excluded from the previous *in vitro* MM [34], has been found to be a significant part of the replication cycle. Lasting approximately 30 h, it is longer than the eclipse phase for influenza A virus and HIV infections in humans (4–24 h) [10, 37]. Although the eclipse phase is included in existing MMs of EBOV-infected animals, its duration has never been estimated, and the assumed values used in these studies were considerably shorter than the value we identify here [12, 14]. Moreover, the observation that the length of the eclipse phase follows an Erlang distribution is contrary to these previous MMs, where it has been represented more simply as an exponentially distributed time. These MMs also fix the value of the decay rate of infectious virus to ensure that other parameters remain identifiable [12]. Here, the robust estimate of this decay rate demonstrates the benefit of performing a mock yield assay. Existing MMs of *in vivo* EBOV infection in humans and non-human primates provide considerably shorter estimates of the infection cycle (12.5–15.3 h) compared to the estimate of 114 h (*τ*_*E*_ + *τ*_*I*_) obtained here [12, 15]. Such a difference is likely attributed to the inclusion of an implicit immune response in these *in vivo* models, thereby accounting for the enhanced clearance of infected cells by immune cells, such as CD8^+^ T cells [38]. This also explains why a faster viral decay rate can be expected *in vivo*, and subsequently why estimates of the basic reproduction number are greater here than those obtained from *in vivo* MMs (5.96-9.01) [12, 15]. It remains to be determined whether Vero cells are representative of the cells targeted by EBOV *in vivo*, but by understanding EBOV replication in Vero cells, we have a foundation from which more complex cell culture models might be developed.

In addition to virus measurements, previous studies have included susceptible and infected cell measurements to fully parameterize the MM and obtain robust estimates of the viral kinetics parameters [19, 39]. We initially set out to obtain a more diverse data set that also included the kinetics of dead cells and intracellular RNA over the course of infection, but encountered unexpected challenges. To quantify cell viability, we treated infected monolayers at various times with Trypan blue, which stains cells that have lost the ability to exclude dye. Unfortunately, we were unable to associate this marker of cell death to a stage of the viral lifecycle in our MM without making additional assumptions. Ultimately, when we extended the MM to include these data, the newly introduced parameters were dependent on these assumptions, and the extracted values of the original parameters were largely unaffected (S1 Appendix). To determine intracellular viral kinetics, the supernatants from infected cell cultures were removed and the remaining monolayers were washed and trypsinized for quantification via TCID_50_ assay and RT-qPCR. These samples showed a high level of EBOV RNA and TCID_50_ as early as 4 hours post-infection, which remained at a constant level up to 1 day post-infection, but rose thereafter (S1 Appendix). Additionally, the ratio of RNA-to-TCID_50_ resembled the ratio observed in the supernatant. Thus, these measurements likely reflect the large amount of cell-associated virions that remained after washing, effectively obscuring the intracellular RNA signal.

While a highly-controlled *in vitro* system was necessary to achieve our precise characterization of the EBOV infection kinetics, the applicability of these results to a clinical situation is not immediately obvious, and represents a serious limitation of the study. Nevertheless, our findings have some relevance to understanding the EBOV infection *in vivo*. EBOV initially replicates within macrophages and dendritic cells in subcutaneous and submucosal compartments, but dissemination in the blood results in the infection of multiple organs throughout the body [40]. Many different cell types are infected with varying susceptibility to infection, as well as varying levels of viral replication. While the infection unfolds, EBOV blocks IFN production early on [6, 41]. In this sense, studying the infection of Vero cells—which are IFN-deficient—narrowly models the infection of one type of epithelial cell during the early stages of an EBOV infection *in vivo*.

Vero cells serve as a standard host cell for replication and are widely used for testing antivirals *in vitro* [42], as well as in the development of viral vaccines [43–45]. Mathematical modelling of EBOV infections *in vitro* using Vero cells has relevance to such applications, particularly in the study of emerging therapeutics. While we provided a quantitative depiction of extracellular infection by EBOV as a valuable first step, we envisioned that the MM could be extended to include intracellular viral RNA kinetics had the appropriate data been collected. Such multiscale modelling approaches have been used to provide insight into virus growth and also to the understanding of direct-acting antivirals [46–49]. We hope that these experiences might help guide future efforts to obtain informative cell and intracellular data.

As an alternative antiviral strategy, there has been renewed interest in pursuing defective interfering particles (DIPs) [50] of highly pathogenic viruses. A DIP is a viral particle that contains defective interfering RNA (DI RNA), which can be a shortened version of the parent genome that renders a DIP replication-incompetent on its own (because it may lack the gene for an essential viral component such as viral polymerase), but also elicits virus-interfering properties. Within a cell co-infected by both DIPs and virus, the DI RNA has a replicative advantage over the full-length RNA and outcompetes it to produce more DIPs than virus progeny, effectively reducing the infectious virus yield. EBOV DI RNA has been detected [51] but much remains to be understood. Like with any other antiviral, MMs can be used to determine the efficacy and mechanism of action of candidate DI RNAs, and to explore the impact of dose and timing [15]. In particular, our estimates of EBOV infection kinetics parameters are directly applicable to future mathematical modelling of the interactions between EBOV and EBOV DIPs *in vitro*. Our estimated EBOV infection parameters may also describe certain aspects of EBOV DIP infection. For example, since DIPs have the same viral proteins and capsid as virions, they would infect cells with the same infection rate constant, *β*. Since DIPs also piggyback on the virus’ replication cycle, we might expect the same eclipse and infectious phase lengths (*τ*_*E*_, *τ*_*I*_) in a DIP and virus co-infected cell.

In summary, the MM described here characterizes the replication cycle of EBOV in a quantitative manner that will be beneficial for those creating *in vitro* models to aid the development of antivirals and vaccines. We have made use of a valuable set of *in vitro* results, carefully considering the structure of the MM in order to maximize the information we can extract from them.

## Materials and methods

### Cells and virus

Vero C1008 cells (ECACC Cat. No.85020206) were obtained from Culture Collection, Public Health England, UK. Vero C1008 cells were maintained in Dulbecco’s minimum essential media supplemented with 10% (v/v) foetal calf serum, 1% (v/v) L-glutamine and 1% (v/v) penicillin/streptomycin (Sigma). For experimental purposes, the foetal calf serum concentration was reduced to 2% (v/v).

Ebola virus *H. sapiens*-tc/COD/1976/Yambuku-Ecran, hereafter referred to as EBOV was used in all studies. This virus, previously known as EBOV “E718” [52] was supplied by Public Health England. Passage 5 material was used to infect Vero C1008 cells. Virus was harvested on day 5 post-inoculation and titrated to produce a working stock at 10^7^ TCID_50_/mL.

### Quantification of virus

EBOV was titrated in 96-well plates using the endpoint fifty percent tissue culture infectious dose (TCID_50_) assay [53]. Briefly, virus was ten-fold serially diluted in 96 well plates of Vero C1008 cells. After one week of incubation at 37°C/5% CO_2_, all wells were observed under the microscope and scored for presence or absence of cytopathic effects. The 50% endpoint was then calculated using the method of Reed & Muench [54]. RNA extractions were performed using the QiAMP Viral RNA Mini Kit (Qiagen, UK). Two 50 μL elutions were performed for each sample to increase the volume available for RT-PCR.

The genetic material of EBOV was quantified using the RealStar^®^ Filovirus Screen RT-PCR Kit (Altona diagnostics, Country) following the instructions of the manufacturer. This assay has been performed many times against a standard curve of plasmid containing the L gene from EBOV. The number of genomes can be estimated from the Ct values as described in Eq (3). In this context, the number of genomes might consist of incomplete negative sense RNA molecules encoding this sequence of the L gene. However, we do not believe that these will be common (<5%) based upon observations made with next generation sequencing (paper in preparation). MOI 5 experiments were analysed using a BIORAD CFX Connect—Real Time System, while samples for the remaining MOIs were analysed using a QuantStudio 7 Flex Real-Time PCR System. Signal from control RNA was compared between experiments and machines and we found no evidence of differences. The parameters of the calibration curve required to convert Ct values to total virus used samples from the MOI 5 experiments.

### Infections

Twenty-four-well plates were seeded with Vero C1008 cells at 10^5^ cells/mL. EBOV was added at MOIs of either 5, 1, or 0.1. Vero cells were grown to 90% confluence for all infections. The cell culture medium was not changed during the experiment and all cultures reached confluence within 24 h (S1 Appendix). At pre-determined intervals post-infection samples were taken by aspiration of supernatant from wells. Samples were stored at −80°C prior to enumeration by TCID_50_ assay and RNA extraction for PCR. Note that the RNA from the MOI 5 infection was omitted from further analysis due to inconsistencies in the peak viral RNA, compared to the MOI 1 and 0.1 infections (S1 Appendix, Fig. A). The viability of Vero cells in the absence of infection is not known under these conditions, however, we have observed these cells for 168 h at 24 h intervals and observed only occasional cells that can be stained with the viability stain Trypan blue.

### Mock yield or infectivity decay assay

EBOV was added to twenty-four-well plates at a final estimated density of 5 × 10^5^ TCID_50_. At pre-determined intervals post-infection samples were taken by aspiration of supernatant from wells. Samples were stored at −80°C prior to enumeration by TCID_50_ assay and RNA extraction for PCR.

### Construction of the standard RT-qPCR curve

**Fig 3:**
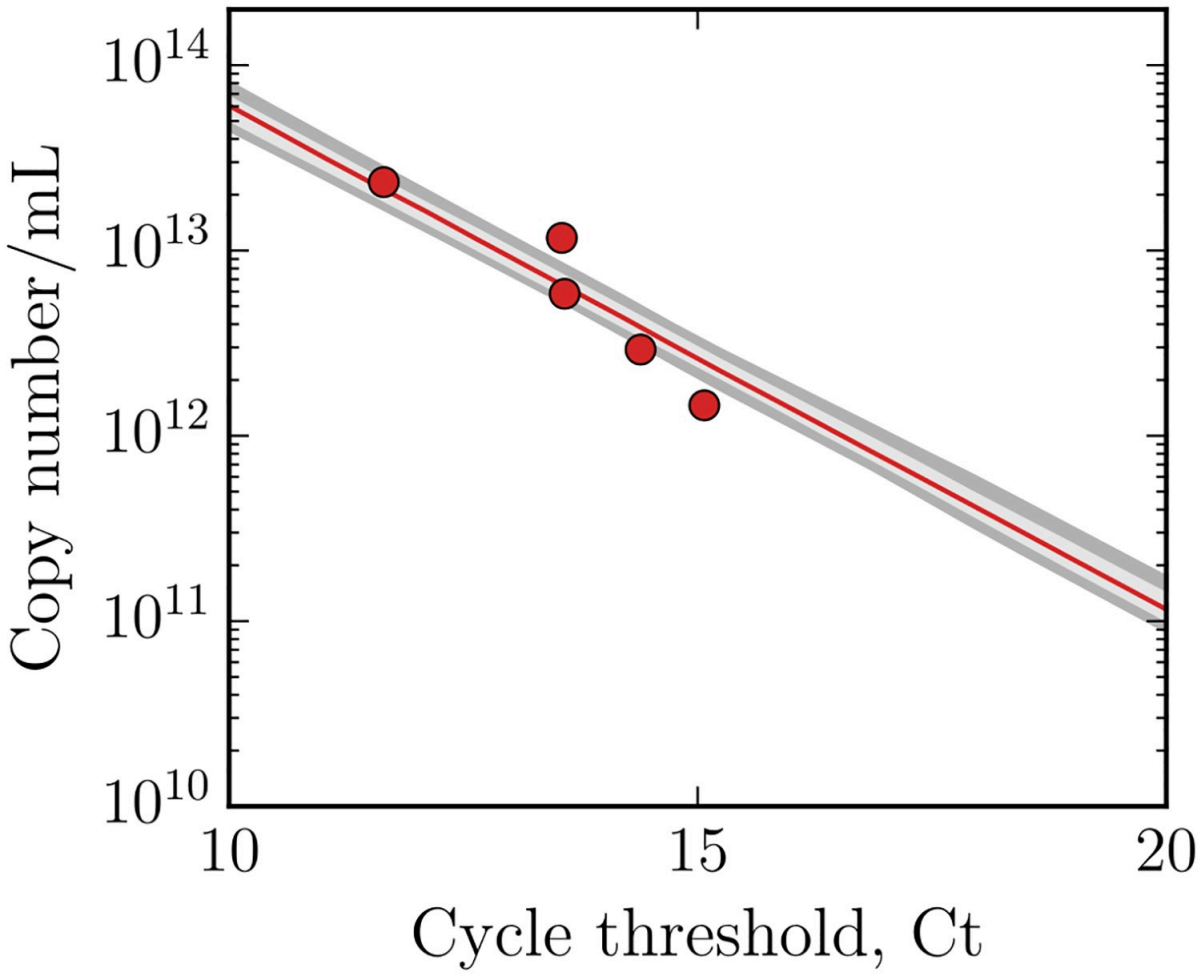
Standard RT-qPCR curve. Cycle threshold values (Ct) were converted to total virus (copy/mL) using the above calibration curve, where the parameters of the curve were estimated as a part of the analysis. The lines represent the pointwise median bracketed by 68% (light grey) and 95% (dark grey) CR.

The concentration of viral genome copies (copy/mL) in a standard sample *i* (*V*_STD,*i*_) and the number of doubling RT-qPCR cycles (*C*_*t*,STD,*i*_) required for this concentration of copies to reach an arbitrarily fixed, chosen threshold concentration (*Q*_*t*_), are linked by the equation

$$\begin{array}{cc} Q_{t} & =V_{\text{STD},i}\;{\left(2\varepsilon \right)}^{C_{t,\text{STD},i}}\\ \ln(V_{\text{STD},i}) & =\underset{y\text{-intercept}}{\underset{︸}{\ln(Q_{t})}}-\underset{\text{slope}}{\underset{︸}{\ln(2\varepsilon )}}\;C_{t,\text{STD},i} \end{array}$$ (2)

where *ε* is the efficacy of the RT-qPCR doubling, which should ideally be equal to one (i.e., exactly doubles at each cycle) but can vary about this value. In constructing the standard curve, we took five standard samples (*V*_STD,*i*=1…5_) with known copy concentrations (via their mass) and determined their corresponding *C*_*t*,STD,*i*_. These data are shown in Fig 3.

### Conversion of sample RT-qPCR *C*_*t*_ values into *V*_tot_

In quantifying the concentration of total virus, *V*_tot_ (copy/mL), in the extracellular virus samples collected from infection experiments, Eq (2) was used as follows

$$\begin{array}{c} \ln\left({V_{\text{tot}}}_{,i}\right)=\ln\left(Q_{t}\right)-\ln\left(2\varepsilon \right)\;C_{t,\text{sample},i}\equiv F\left(C_{t,\text{sample},i}\right) \end{array}$$ (3)

where *V*_tot,*i*_ is the concentration of copies in sample *i*, given its RT-qPCR-determined *C*_*t*,sample, *i*_ value. Here, ln(*Q*_*t*_) and ln(2*ε*) are two parameters to be estimated as part of the MCMC parameter estimation process described later in this section. As different values for these two parameters are sampled in the MCMC process, the total virus concentration data points vary. The variation in the conversion is denoted by error bars on each total virus data point in Fig 1E–1G.

### Mock-yield assay model

Loss of virus infectivity or loss of viral genome integrity over time typically follows an exponential decay [22]. As such, the mock-yield (MY) or infectivity decay assay can be captured via *V*(*t*) = *V*_0_ e^−*ct*^, such that the experimental MY data are expected to follow

$$\begin{array}{cc} \ln(V_{\text{inf}}\left(t\right)) & =\ln\left(V_{\text{inf},0}^{\text{MY}}\right)-c_{\text{inf}}\;t \end{array}$$ (4)

$$\begin{array}{cc} \ln(V_{\text{tot}}\left(t\right)) & =\ln\left(V_{\text{tot},0}^{\text{MY}}\right)-c_{\text{tot}}\;t \end{array}$$ (5)

where *V*_inf_(*t*) and *V*_tot_(*t*) are the concentrations of infectious (TCID_50_/mL) and total (copy/mL) virus after an incubation of time *t* under the same conditions used during the infection experiments, given the EBOV rate of loss of infectivity (*c*_inf_) or integrity (*c*_tot_), and initial concentrations, $V_{\text{inf},0}^{\text{MY}}$ and $V_{\text{tot},0}^{\text{MY}}$. These data are shown in Fig 1D and 1G.

### Simulated infections and parameter estimation

In estimating the MM parameters, the following experimental data were considered simultaneously: the RT-qPCR standardized curve (5 data points), the MY assays (24 data points: 4 time points in triplicate for *C*_*t*_ and *V*_inf_), and three infection assays at MOI of 5 (24 data points: 6 time points in duplicate for *C*_*t*_ and *V*_inf_), MOI of 1 (54 data points: 9 time points in triplicate for *C*_*t*_ and *V*_inf_), and MOI of 0.1 (53 data points: 9 time points in triplicate for *C*_*t*_ and *V*_inf_, minus one contaminated sample in *C*_*t*_).

Eq (2) was used to capture the RT-qPCR standard curve, and its agreement with the 5 experimental data points was computed as the sum-of-squared residuals (SSR)

$$\begin{array}{c} {\text{SSR}}^{\text{STD}}=\frac{\sum_{i=1}^{5}{\left[F\left(C_{t,\text{STD},i}\right)-\ln\left(V_{\text{STD},i}\right)\right]}^{2}}{\sigma_{V_{\text{tot}}}^{2}}\;, \end{array}$$

where $\sigma_{V_{\text{tot}}}^{2}$ is the variance, or squared of the standard error, in experimentally measured *V*_tot_, which will be discussed in more details below. Eqs (4) and (5) were used to capture the MY experiment, performed in triplicate, and sampled at 4 time points, for each of *C*_*t*_ and *V*_inf_, and agreement was computed as

$$\begin{array}{c} {\text{SSR}}^{\text{MY}}=\frac{\sum_{i=1}^{12}{\left[\ln\left(V_{\text{inf},0}^{\text{MY}}\right)-c_{\text{inf}}\;t_{i}-\ln\left(V_{\text{inf}}\left(t_{i}\right)\right)\right]}^{2}}{\sigma_{V_{\text{inf}}}^{2}}\\ +\frac{\sum_{i=1}^{12}{\left[\ln\left(V_{\text{tot},0}^{\text{MY}}\right)-c_{\text{tot}}\;t_{i}-F\left(C_{t,i}\right)\right]}^{2}}{\sigma_{V_{\text{tot}}}^{2}} \end{array}$$

Finally, MM Eq (1) was used to reproduce the infection experiments, performed in triplicate, and at 3 different MOIs (5, 1, and 0.1), and quantified via both TCID_50_ and RT-qPCR. In reproducing the infections, initial conditions (at *t* = 0) were such that *T*(0) = 1, $E_{i=1,...,n_{E}}\left(0\right)=I_{j=1,...,n_{I}}\left(0\right)=0$, and the initial infectious and total virus concentrations for the 3 MOIs were computed as:

$$\begin{array}{cc} V_{\text{inf}}\left(0\right) & =V_{\text{inf},0}^{\text{INF}}\times \text{MOI}\\ V_{\text{tot}}\left(0\right) & =V_{\text{tot},0}^{\text{INF}}\times \text{MOI} \end{array}$$

where MOI was either 5, 1 or 0.1, and ($V_{\text{inf},0}^{\text{INF}}$, $V_{\text{tot},0}^{\text{INF}}$) are 2 parameters to be estimated. Agreement between MM Eq (1) and experimental infection data was computed as

$$\begin{array}{c} {\text{SSR}}^{\text{INF}}=\sum_{\text{MOI}=[5,1,0.1]}\frac{\sum_{i}{\left[\ln(V_{\text{inf}}^{\text{MM}}\left(t_{i}\right)-\ln\left(V_{\text{inf}}\left(t_{i}\right)\right)\right]}^{2}}{\sigma_{V_{\text{inf}}}^{2}}\\ +\frac{\sum_{i}{\left[\ln(V_{\text{tot}}^{\text{MM}}\left(t_{i}\right)-F\left(C_{t,i}\right)\right]}^{2}}{\sigma_{V_{\text{tot}}}^{2}} \end{array}$$

where $\sigma_{V_{\text{inf}}}^{2}=0.1$ and $\sigma_{V_{\text{tot}}}^{2}=0.1$ correspond to the variance in ln(*V*_inf_) and ln(*V*_tot_), respectively, estimated as the variance of the residuals between the 2 to 3 replicates of ln(*V*_inf_) or ln(*V*_tot_) measured at each time point and their corresponding mean, across all (STD, MY, and INF) experimental data collected.

A total of 15 parameters—6 parameters associated with experimental conditions (ln(*Q*_*t*_), ln(2*ε*), $\ln(V_{\text{inf},0}^{\text{MY}})$, $\ln(V_{\text{tot},0}^{\text{MY}})$, $V_{\text{inf},0}^{\text{INF}}$, $V_{\text{tot},0}^{\text{INF}}$) and 9 parameters more closely associated with EBOV infection kinetics (*c*_inf_, *c*_tot_, *p*_inf_, *p*_tot_, *β*, *τ*_*E*_, *τ*_*I*_, *n*_*E*_, *n*_*I*_)—were estimated (Table 1) from 160 experimental data points using the python MCMC implementation phymcmc [55], a wrapping library for emcee [56]. Posterior probability likelihood distributions (PostPLDs) were obtained based on the parameter likelihood function

$$\begin{array}{c} \ln\left(L\left(\vec{p}\right)\right)=-\frac{1}{2}[{\text{SSR}}^{\text{STD}}\left(\vec{p}\right)+{\text{SSR}}^{\text{MY}}\left(\vec{p}\right)+{\text{SSR}}^{\text{INF}}\left(\vec{p}\right)] \end{array}$$

and the assumption of linearly uniform or ln-uniform priors, where $\vec{p}$ is the 15-parameter vector.

**Table 1:** Estimated parameters of EBOV infection *in vitro*.

Parameter	Mode [95% CR]
Infectiousness, *β* ($\frac{\text{mL}}{{\text{TCID}}_{50}\cdot h}$)	10^−6.48 [−6.7, −6.3]^
Eclipse phase length, *τ*_*E*_ (h)	30.5 [26, 37]
Number of eclipse compartments, *n*_*E*_	13 [8, 23]
Infectious phase length, *τ*_*I*_ (h)	83.2 [64, 95]
Number of infectious compartments, *n*_*I*_	14 [3, 85]
Infectious virus production rate, *p*_inf_ ($\frac{{\text{TCID}}_{50}}{\text{cell}\cdot h}$)	10^1.12 [1, 1.3]^
Total virus production rate, *p*_tot_ ($\frac{\text{RNA}}{\text{cell}\cdot h}$)	10^6.46 [6.3, 6.7]^
Rate of loss of infectious virus, *c*_inf_ (/h)	0.0614 [0.055, 0.068]
Rate of virus degradation, *c*_tot_ (/h)	0.00817 [0.0035, 0.013]
Initial infectious virus inoculum, $V_{\text{inf},0}^{\text{INF}}$ ($\frac{{\text{TCID}}_{50}}{\text{mL}}$)	10^5.39 [5.3, 5.5]^
Initial total virus inoculum, $V_{\text{tot},0}^{\text{INF}}$ ($\frac{\text{RNA}}{\text{mL}}$)	10^11.5 [11, 12]^
MY initial infectious virus inoculum, $\ln(V_{\text{inf},0}^{\text{MY}})$	13.7 [13, 14]
MY initial total virus inoculum, $\ln(V_{\text{tot},0}^{\text{MY}})$	28.2 [28, 29]
Standard RT-qPCR curve *y*-intercept, ln(*Q*_*t*_)	37.8 [37, 39]
Standard RT-qPCR curve slope, ln(2*ε*)	0.613 [0.57, 0.66]
Basic reproductive number, *R*_0_	10^2.77 [2.6, 3]^
Infectious burst size, *p*_inf_ *τ*_*I*_ ($\frac{{\text{TCID}}_{50}}{\text{cell}}$)	10^3.04 [3, 3.1]^
Infecting time, *t*_inf_ (h)	10^0.335 [0.21, 0.43]^

## Supporting information

S1 Appendix Kinetics of cell-associated virus and cell viability. (PDF)
