# Supplementary material for: Quantification of Ebola virus replication kinetics in vitro
Source: PLoS Comput Biol. 2020 Nov 2;16(11):e1008375. doi: 10.1371/journal.pcbi.1008375 (PMC7660928; doi:10.1371/journal.pcbi.1008375)
Supplement: S1 Appendix — (PDF) [file pcbi.1008375.s001.pdf]

Supporting Information S1:

Quantification of Ebola virus replication kinetics in vitro

Laura E. Liao, Jonathan Carruthers, Sophie J. Smither, CL4 Virology Team, Simon A. Weller, Diane Williamson, Thomas R. Laws, Isabel García-Dorival, Julian Hiscox, Benjamin P. Holder, Catherine A. A. Beauchemin, Alan S. Perelson, Martín López-García, Grant Lythe, John Barr, Carmen Molina-París

October 8, 2020

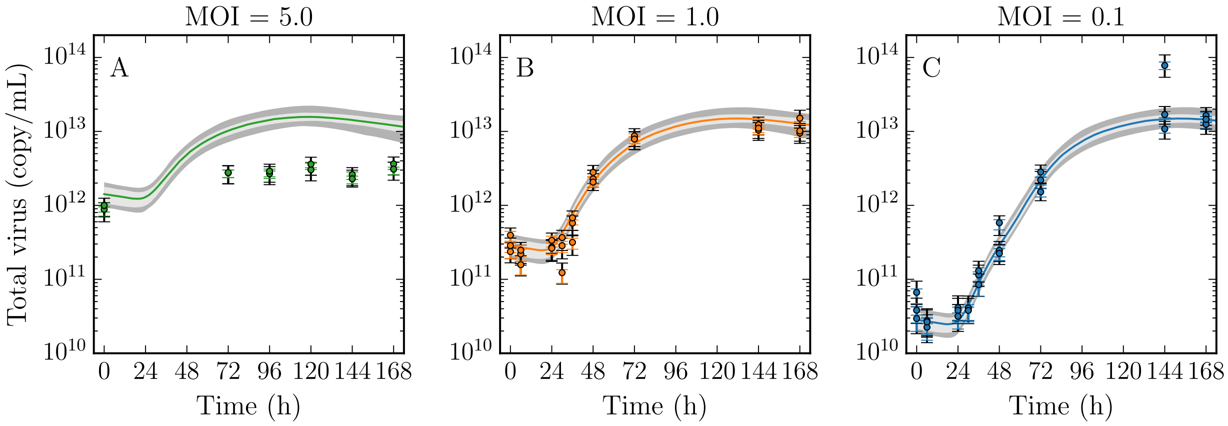

Figure A: **Total virus from MOI 5 infection.** The total virus concentration from the MOI 5 infection (A) was omitted from the analysis due to an order of magnitude difference in the peak concentration when compared to the MOI 1.0 and 0.1 infections (B, C). It was unclear whether this discrepancy was biologically meaningful or due to systematic experimental error.

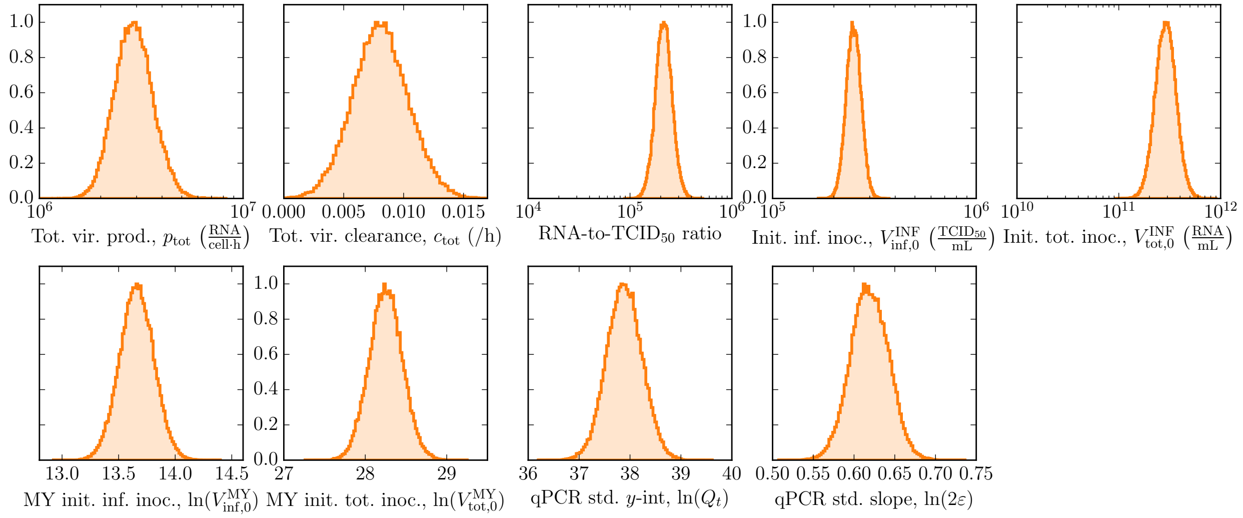

Figure B: **Estimated parameter distributions of EBOV infection in vitro.** Posterior probability likelihood distributions describing the total virus, mock yield, and calibration curves.

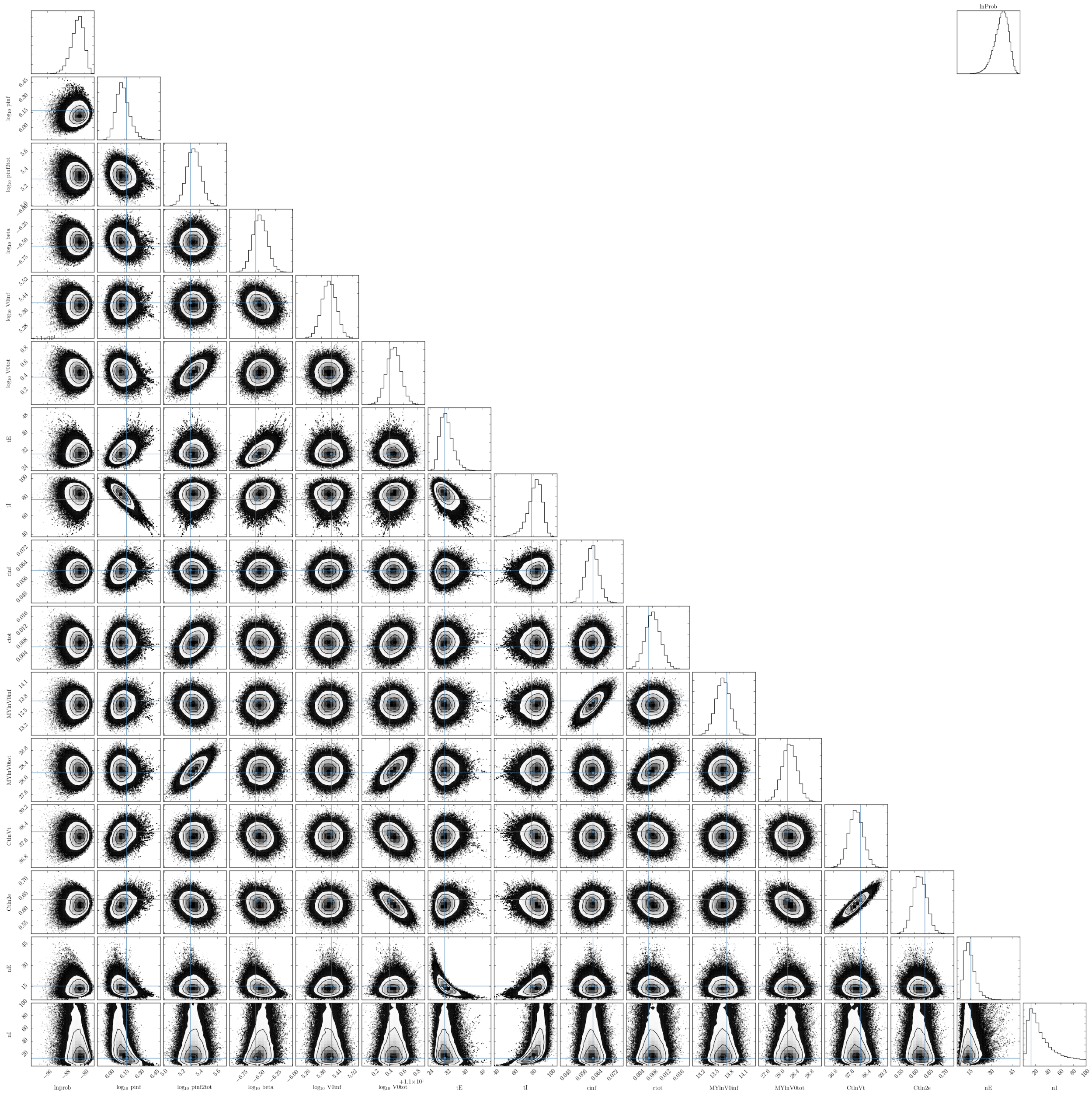

Figure C: **Paired parameter posterior likelihood distributions.** Two-parameter PLDs for each of the 600,000 MCMC-accepted parameter sets are shown. Although mild correlations were observed, they were not important given that narrow PLDs were obtained. The majority of correlations were a consequence of performing linear regression to the mock yield and standard qRT-PCR curve, resulting in correlations between the (slope, y-int) parameter pairs (c_{inf}, $\ln(V^{\mathrm{MY}}_{\mathrm{inf},0})$ and ($\ln(2\varepsilon)$, $\ln(Q_t)$), respectively. Since different linear fits to the standard curve impacts the conversion of Ct to RNA, correlations between the total virus parameters are also observed as a result, e.g., ($\ln(2\varepsilon)$, $V^{\mathrm{INF}}_{\mathrm{tot},0}$), ($\ln(V^{\mathrm{MY}}_{\mathrm{tot},0})$, p_{tot}), ($\ln(V^{\mathrm{MY}}_{\mathrm{tot},0})$, $V^{\mathrm{INF}}_{\mathrm{tot},0}$). Other correlations are observed such as (p_{inf}, τ_I), (n_E, τ_E), (n_I, t_I).

1 Kinetics of Cell-associated Virus

Two MOI 5 infection experiments were performed with cells grown to 90% and 70% confluence in Expt 1 and Expt 2, respectively. In Expt 1 (Figure D, panels A, B; green), infectious and total virus concentrations were determined from the supernatant of the in vitro assay at times late in the infection (0 and 72–168 hours post-infection). In Expt 2, earlier and much more frequent sampling (every 2 hours from 0–22 hours post-infection) was performed to determine the infectious and total virus concentrations in the supernatant (Figure D, panels A, B; yellow). After supernatant removal, additional samples were taken where cell monolayers were washed with PBS, trypsinized and scraped with a pipette tip for total and infectious virus quantification (Figure D, panels C, D).

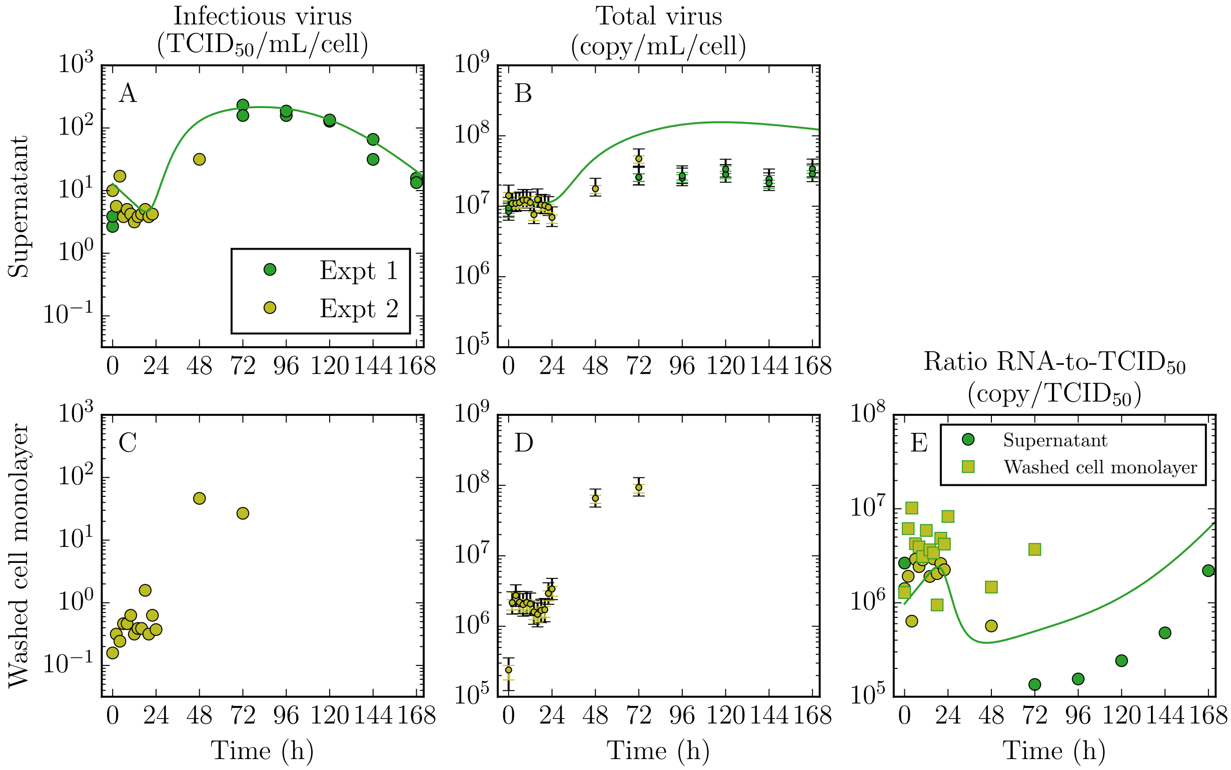

Figure D: **Kinetics of MOI 5 infection.** In two MOI 5 infection experiments, the infectious and total virus concentrations in the supernatant (A, B) of the in vitro assay were quantified at primarily late times (Expt 1) or early times (Expt 2). In addition, Expt 2 quantified the virus concentrations from washed and trypsinized cell monolayers that remained after removal of the supernatant (C, D). The ratio of RNA-to-$TCID_{50}$ in the supernatant is compared to that in the washed cell monolayers in E. Note that these data have been normalized to the number of cells per well (10^5 cells). The lines represent the simulated time course from our MM that corresponds to the set of parameters with the maximum log-likelihood. The variability from converting Ct values to total virus (copy/mL) is shown by two error bars on each total virus data point, denoting the 68% (same colour) and 95% (black) CR.

We expected that the virus concentrations from the washed cell monolayers would correspond

to intracellular virus concentrations (Figure D, panels C, D). We speculate that the intracellular virus signal was instead obscured by cell-associated virus for the following reasons. Firstly, a very high level of total virus was detected even as early as 0–2 hours post-infection, before significant viral replication was expected to occur. This was accompanied by a very high level of infectious virus which, if we were to believe were due to intracellular virions, is counter to the fact that virions uncoat and release viral RNA once they enter the cell and would not register as infectious virions in a TCID_{50} assay. Lastly, in Figure D (panel E), we compared the ratio of RNA-to-TCID_{50} in the supernatant (circles) to that from the washed cell monolayers (squares). The ratio at early times from the washed cell monolayers resembled the ratio in the supernatant. If the washed cell monolayers samples were to reflect the intracellular virus levels, we would expect this ratio to be much higher (more RNA than TCID_{50} within a cell). A likely possibility is that these data reflect the kinetics of cell-associated virions, which are virions that remained attached to the cell membrane even after washing.

Under the interpretation that these data reflect cell-associated virus kinetics, the high levels of virus at early times are likely virions that adsorbed to the cell membrane. A small jump in virus concentration between 0–2 hours post-infection can be observed, perhaps showing that adsorption is still occurring, until it reaches a steady state by 4 hours post-infection. Once the infected cell comes out of the eclipse phase, by 48 hours post-infection, the ratio of RNA-to-TCID_{50} rises. These data at late times (48, 72 hours post-infection) may no longer be dominated by the cell-associated virus signal, and could contain information on the level of intracellular virus. We did not pursue further mathematical modelling due to the lack of intracellular virus data, beyond these two data points.

Our final analysis only included the infectious virus concentration from Expt 1. There was insufficient information to justify whether the data from Expt 1 and 2 should be combined. We could not verify whether there was consistency in the peak virus concentration, the timing of the rise, or the difference between peak and early virus levels since Expt 2 lacked frequent sampling late enough in the infection to capture such features. As previously mentioned, the total virus concentration from Expt 1 was also omitted. Ultimately, we found that omission of these data did not hamper our ability to robustly extract the viral infection kinetics parameters.

2 Kinetics of Cell Viability and Infection

2.1 Methods

Following the removal of the supernatant for virus quantification, three wells (two for the MOI = 5 experiment) at each time point were stained with 5% Trypan blue for ≥ 2 min, then visualised and photographed at fixed magnification using a Leica DMIRB microscope and the Leica Application Suite v4.9 software.

The total number of stained cells in each image was counted directly since these were generally unambiguously identifiable. The total number of cells per image was estimated by counting intact cells within 5–10 randomly placed windows, $1/64^{\text{th}}$ the area of the image, and computing an average (Figure E). Windows in which cell boundaries could not be reliably identified, due to image artifacts or optical effects, were rejected. The mean and standard deviation of each quantity over the replicates was used for analysis.

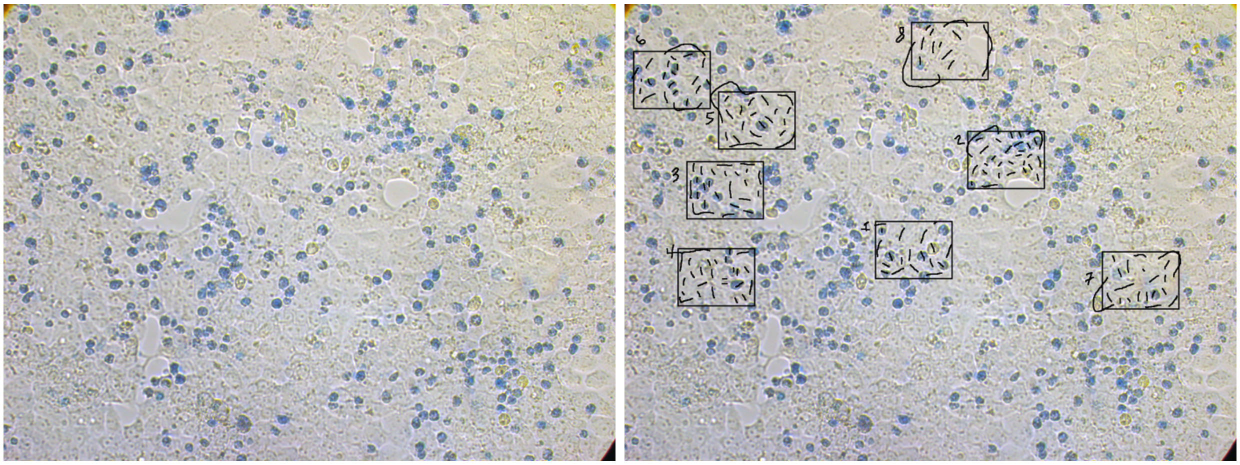

Figure E: Representative image — one of the two MOI = 5 images at 168 h — of Trypan blue stained cells in infected monolayers (left), and the counting method used to estimate total number of cells per image (right). Random windows chosen for cell counting were rejected and re-selected if the cells boundaries were not clearly visible. Trypan–blue-stained cells were counted directly.

2.2 Analysis

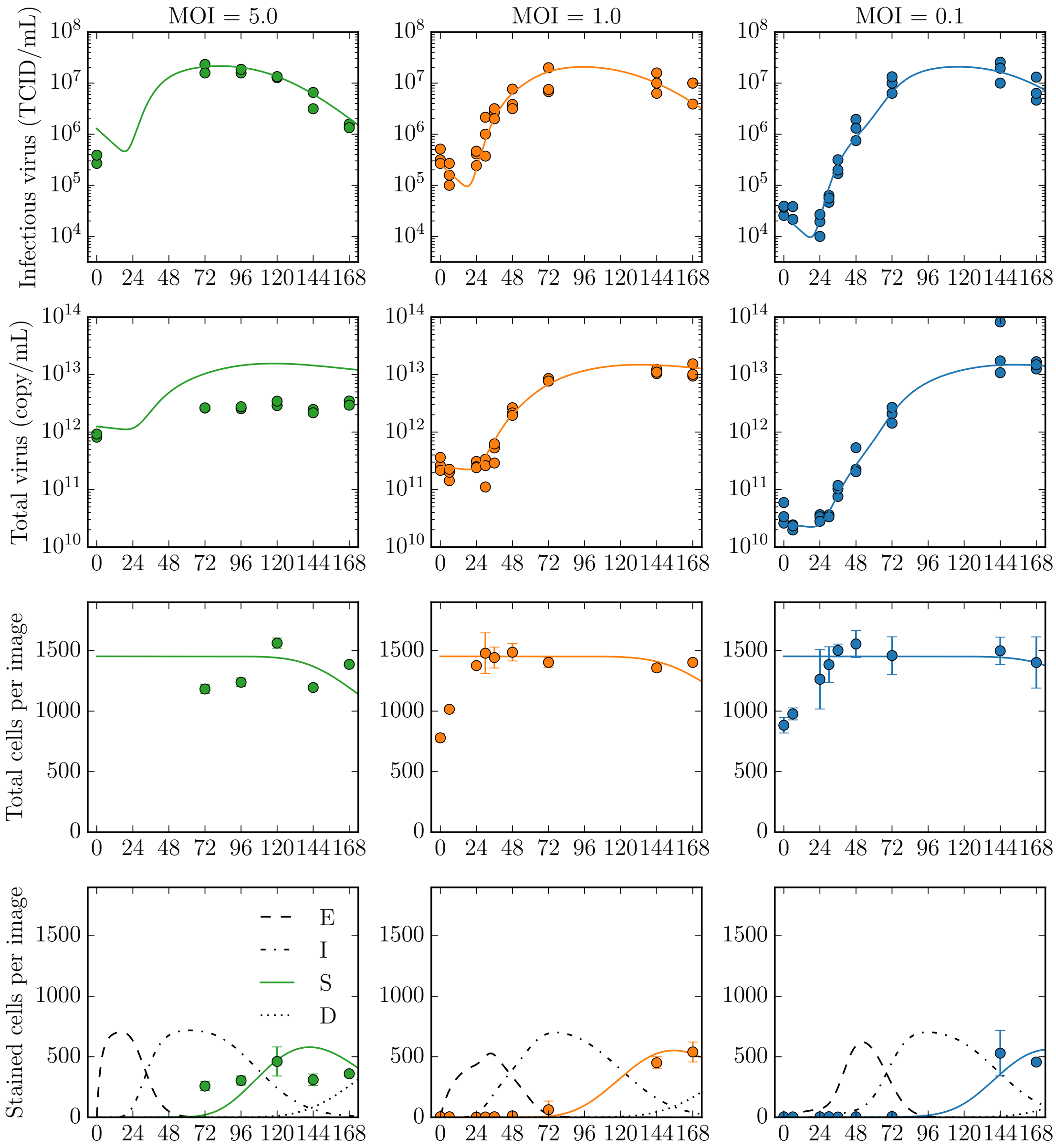

Figure F: Simultaneous fit of viral kinetics data and cell viability kinetics with extended model (see text). To simulate the infection dynamics of the number of cells in each image, we started the infection with $N_{\text{image}} = 1450$ target cells in the extended model, and rescaled the viral production rates (e.g., $p_{\text{inf}} \frac{N_{\text{well}}}{N_{\text{image}}}$) in order to model the virus concentrations in the entire well. The total cells per image in the model was given by $T + \sum_{1}^{n_E} E_i + \sum_{1}^{n_I} I_j + S$.

The total cell counts per image revealed a very stable population of intact cells (approximately 1450 cells per image for all three MOI experiments), following a brief period of proliferation in the first day post-infection (Figure F, Total cells per image). In the MOI = 1 experiment, the percent of cells that were stained — Trypan blue marks cells that have lost the ability to actively exclude the dye — was low ($\sim 5\%$) at 72 h post-infection but rose to approximately 30% by one week post-infection. In the high MOI experiment, 20–30% of cells were stained by the first measured time point (72 h) and that level was maintained throughout the experiment, while cells infected at low MOI remained unstained at 72 h, but were stained at the same 20–30% level at the next measured timepoint, three days later.

Despite a nearly constant population size over time, many cell cultures imaged at later times had large holes in the otherwise regular monolayer. These seemingly contradictory observations suggest that, near the end of the one week experiment, a small number of cells disintegrated completely and the monolayer surface tension created larger holes in their absence.

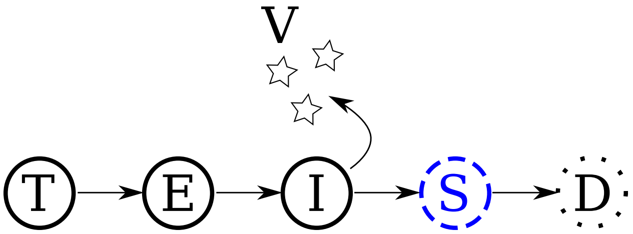

Figure G: Extended Ebola infection model in which Trypan-blue stained cells (S) represent an early phase of cell death prior to their disintegration into uncountable debris (D).

To simulate these cell viability kinetics in tandem with our infection model (presented in the main text) we assumed that once infected cells cease viral production, they enter a relatively long-lasting phase where their membrane is permeable to Trypan blue and they become stained (S) cells (Figure G). As the stages of cell death progress, stained cells subsequently disintegrate to debris (D) and cannot be counted. Representing the stained phase with a sequence of n_{S} equations to obtain Erlang-distributed timings, i.e.,

$$\frac{\mathrm{d}S_1}{\mathrm{d}t} = \frac{n_I}{\tau_I} I_{n_I} - \frac{n_S}{\tau_S} S_1$$

$$\frac{\mathrm{d}S_{j=2,3,\ldots,n_S}}{\mathrm{d}t} = \frac{n_S}{\tau_S} \left(S_{j-1} - S_j\right) ,$$

we found that an average S time of 70 h ($n_{\mathrm{S}} = 10$) was sufficient to reproduce the observed constant total cell count per image over the one week experiment (Figure F, Total cells per image). Finally, to account for the fact that stained cells made up only a modest fraction of the total number of cells per image, even at late times, we allowed for only 50% of the target cells to be susceptible to infection. Under these assumptions, we obtained an adequate fit to the stained cell data for all three MOI experiments (Figure F, Stained cells per image) while holding all but one of the infection parameters (the viral production rate was doubled to account for the halved susceptible population) at their maximum likelihood values (Table 1, main text), thus maintaining agreement with the viral kinetics data.

2.3 Discussion

In our final analysis (main text), we chose to exclude cell data from consideration because, at least in its simplest interpretation, it had no effect on the estimation of the viral kinetics parameters (the only exception being the viral production rate, which could differ by a factor of two, as discussed above). Moreover, any additional information that could be determined about the viability kinetics of EBOV-infected cells would be based solely on assumptions about how the timing of Trypan blue staining fits within the infection timeline. The Trypan blue dye stains cells that can no longer actively exclude it, implying that stained cells are dead. But the onset of this passive permeability with respect to cessation of viral production (the end of the "infectious" phase of infection) is not known, and therefore acts as a hidden parameter (see below). Future resolution of this problem, or the use of a staining method that can be registered to the infection timeline, e.g., immuno-staining, could potentially allow for these cell kinetics data to further constrain the viral kinetics parameters.

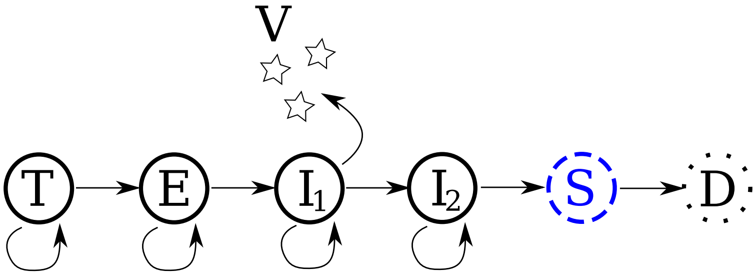

Figure H: Alternative model in which a proliferating "post-infectious" phase (I_2) continuously feeds a quasi–steady-state population of stained cells (S). Tuning the relative lifespans of each population can yield the observed fractional level of the stained population, without the requirement that some target cells are not susceptible to infection.

We were able to obtain slightly better agreement to the cell kinetics data (not shown) with additional model features. Adding logistic proliferation for target and eclipse cells to the above-described model (with a growth rate $r = 0.105\ \mathrm{h}^{-1}$ common to all three MOI experiments) allowed for a very good fit to the first 24 h of cell data, without altering the agreement to the stained cell or viral kinetic data. To avoid the assumption that only half of the target cells are susceptible, an alternative model (Figure H) could be used where one assumes: (i) a post-infectious phase (I_2) in which infected cells no longer produce virus but continue to actively exclude dye, and (ii) that all living cells, including those in the post-infectious phase, proliferate. This results in a model with the following set of equations for the post-infectious phase:

$$\frac{\mathrm{d}I_{21}}{\mathrm{d}t} = \frac{n_{I_1}}{\tau_{I_1}} I_{1n_{I_1}} - \frac{n_{I_2}}{\tau_{I_2}} I_{21} + r\, I_{21} \left(1 - \frac{T + \sum E_j + \sum I_{1j} + \sum I_{2j}}{N_{\max}}\right)$$

$$\frac{\mathrm{d}I_{2j=2,3,\ldots,n_{I_2}}}{\mathrm{d}t} = \frac{n_{I_2}}{\tau_{I_2}} \left(I_{2j-1} - I_{2j}\right) + r\, I_{2j} \left(1 - \frac{T + \sum E_j + \sum I_{1j} + \sum I_{2j}}{N_{\max}}\right),$$

and other model equations adapted accordingly. Under these assumptions the stained (S) cells can form a late-time quasi–steady-state population that is constantly replenished by ongoing proliferation, whose fractional level with respect to the total population can be tuned by the relative rates of entry and exit to/from the S phase. This allows for a better fit to the stained cell data than

that shown in Figure F (Stained cells per image), but requires a re-tuning of infection parameters to maintain agreement with the viral kinetics data. Given the current lack of knowledge about the end stage viability of infected cells, and the ambiguity of the Trypan blue stain, described above, we did not pursue these more complicated models any further.
